# Adverse Hemodynamics in Acute Heart Failure Patients Are Associated with Skeletal Muscle Stress Biomarkers

**DOI:** 10.3390/ijms27114850

**Published:** 2026-05-28

**Authors:** Kamil A. Kobak, Monika Kasztura, Krystian Josiak, Robert Zymliński, Natalia Świątoniowska-Lonc, Waldemar Banasiak, Kinga Węgrzynowska-Teodorczyk

**Affiliations:** 1 Aging and Metabolism Research Program, Oklahoma Medical Research Foundation, Oklahoma City, OK 73104, USA; 2 Department of Food Hygiene and Consumer Health Protection, Faculty of Veterinary Medicine, Wroclaw University of Environmental and Life Sciences, 50-375 Wroclaw, Poland; monika.kasztura@upwr.edu.pl; 3 Department of Cardiology, Center for Heart Diseases, 4th Military Hospital, 50-981 Wroclaw, Poland; kjosiak@4wsk.pl (K.J.); natalia.swiat@o2.pl (N.Ś.-L.); banasiak@4wsk.pl (W.B.); kinga.wegrzynowska@awf.wroc.pl (K.W.-T.); 4 Institute of Heart Diseases, Wroclaw Medical University, 50-556 Wroclaw, Poland; robert.zymlinski@umw.edu.pl; 5 Clinical Department of Cardiology, Faculty of Medicine, Wroclaw University of Science and Technology, 50-370 Wroclaw, Poland; 6 Faculty of Physiotherapy, Wroclaw University of Health and Sport Sciences, 51-612 Wroclaw, Poland

**Keywords:** acute heart failure, skeletal muscle, biomarkers, hemodynamics

## Abstract

Acute heart failure (AHF) causes abrupt hemodynamic disturbances, including reduced forward flow and venous congestion, which may extend beyond the heart and contribute to peripheral organ stress. Skeletal muscle may be particularly vulnerable to these changes, but the relationship between acute hemodynamic status and circulating markers of skeletal muscle stress and regulation remains unclear. We prospectively enrolled 35 men hospitalized with AHF and non-invasively assessed their cardiac index (CI) by impedance cardiography and right atrial pressure (RAP) by echocardiography. Plasma carbonic anhydrase III (CA3), creatine kinase-MM (CK-MM), lactate, myostatin, and follistatin were measured at admission, discharge, and 30 days after discharge. Patients were analyzed according to low CI, defined as CI < 2.2 L·min^−1^·m^−2^; elevated RAP, defined as RAP ≥ 8 mmHg; and combined CI/RAP profiles. CA3 and CK-MM were higher in patients with low CI or elevated RAP and were highest in the low-CI/elevated-RAP profile. CA3 and lactate did not significantly change during follow-up, whereas CK-MM modestly increased at 30 days. Myostatin and follistatin were highest at admission and decreased after clinical stabilization. In this pilot cohort of men hospitalized with AHF, estimated lower perfusion and greater venous congestion were associated with higher circulating markers of skeletal muscle stress, while muscle regulatory myokines declined after stabilization. These findings suggest that skeletal muscle-related biomarkers may reflect peripheral consequences of acute hemodynamic disturbance in AHF and warrant further investigation in larger cohorts.

## 1. Introduction

Recurrent hospitalizations and a progressive decline in physical function are major contributors to impaired quality of life in heart failure (HF) [1,2]. Acute heart failure (AHF), defined by the rapid worsening of HF symptoms, affects approximately 2% of the European population and is associated with high short-term mortality and rehospitalization risk [3,4]. Despite its clinical importance, AHF remains difficult to characterize because of its diverse etiologies, heterogeneous presentation, and complex interplay between hemodynamic and neurohormonal disturbances [5].

AHF is characterized by profound hypoperfusion and venous congestion that injure the peripheral organs and predict worse outcomes [5,6]. Renal, hepatic, and myocardial injury are well described in this setting [6,7], yet the effects on other organs are poorly characterized. Skeletal muscle dysfunction is well described in chronic HF, with loss of mass and strength, fiber type shifts, and reduced oxidative capacity that drive exercise intolerance and adverse prognosis [1,2]. Although muscle health shapes functional recovery, frailty trajectories, and outcomes after hospital discharge [8,9], the effects of acute decompensation in AHF on skeletal muscle are not well described. In particular, it remains unclear how the acute hemodynamic insult of AHF affects skeletal muscle biology and whether these changes persist beyond discharge.

The aim of this study was to test whether adverse hemodynamics in AHF are linked to skeletal muscle stress signals and altered myokine regulators of muscle growth. We hypothesized that lower perfusion and greater venous loading would contribute to higher muscle stress signals and altered myokine patterns. We used noninvasive bedside assessments to characterize perfusion and congestion: cardiac index (CI) served as a proxy for peripheral perfusion, and echocardiographic right atrial pressure (RAP) served as a surrogate for venous congestion. We measured circulating markers associated with skeletal muscle stress and impaired oxidative metabolism, including carbonic anhydrase III (CA3), creatine kinase MM and lactate [10,11,12,13], along with myostatin and follistatin as regulators of muscle growth [14,15,16,17].

## 2. Results

### 2.1. Baseline Characteristics

The baseline characteristics of 35 male patients with AHF are presented in Table 1.

### 2.2. Lower Cardiac Index Is Associated with Higher Skeletal Muscle Stress Biomarkers

We first evaluated whether circulating biomarkers of skeletal muscle stress and regulation were related to standard cardiac indices. No significant associations were observed among CA3, CK-MM, myostatin, or follistatin and systolic function (LVEF), myocardial injury (troponin I), or neurohormonal activation (NTproBNP). Similarly, none of the plasma biomarkers correlated with stroke volume or cardiac output. Because stroke volume and cardiac output SV and CO are influenced by body size and loading conditions, we next examined cardiac index (CI) as an estimate of perfusion adequacy relative to body size. CI correlated inversely with CA3 (r = −0.36) and CK-MM (r = −0.39; both *p* < 0.05), whereas myostatin and follistatin showed no association with CI. According to the Forrester classification, a CI below 2.2 L·min^−1^·m^−2^ indicates peripheral hypoperfusion [18]. We therefore used 2.2 as the threshold to stratify patients by CI (CI < 2.2 vs. ≥2.2; Table 2) into low CI (≤2.2 L·min^−1^·m^−2^; *n* = 15) and normal CI (>2.2 L·min^−1^·m^−2^; *n* = 20).

**Figure 1:**
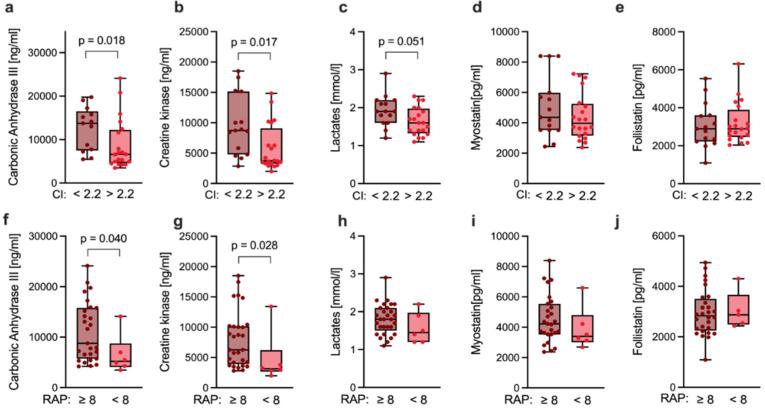
Skeletal muscle stress biomarkers and myokine signals across perfusion (CI) and congestion (RAP) status in acute heart failure. (**a**–**e**) Comparison by cardiac index (CI > 2.2 vs. <2.2 L·min^−1^·m^−2^): (**a**) Carbonic anhydrase III (CA3), (**b**) creatine kinase–MM (CK-MM), (**c**) lactate, (**d**) myostatin. (**e**) follistatin (**f**–**j**) Comparison by right atrial pressure (RAP < 8 vs. ≥8 mmHg): (**f**) CA3, (**g**) CK-MM, (**h**) lactate, (**i**) myostatin, and (**j**) follistatin. Boxes denote interquartile ranges; central lines indicate medians; whiskers show minimum and maximum values; individual data points are displayed. Group comparisons used two-sided Mann–Whitney U tests. CI, cardiac index; RAP, right atrial pressure; CA3, carbonic anhydrase III; CK-MM, creatine kinase–MM.

The groups did not differ in age, heart rate, NTproBNP, troponin, oxygen saturation, LVEF, or estimated RAP. Patients with low CI had lower arterial blood pressure, lower BMI, lower hemoglobin, and worse kidney function (higher creatinine, lower eGFR). Serum CA3 and CK-MM concentrations were higher in the low-CI group compared with the normal CI group (*p* = 0.018 and *p* = 0.017, respectively), with a trend toward higher lactate (*p* = 0.051) (Figure 1a–c). Myostatin and follistatin did not differ between CI categories (Figure 1d,e). Because renal function and body composition differed between CI groups and may influence circulating skeletal muscle stress-related biomarkers, we performed exploratory multiple linear regression analyses in the entire cohort. The model included eGFR, BMI, systolic blood pressure, and hemoglobin. eGFR and BMI were significant predictors of both CK-MM and CA3. Lower eGFR and lower BMI were significant predictors of higher CK-MM concentrations (eGFR: standardized β = 0.20, *p* < 0.001; BMI: standardized β = 0.16, *p* < 0.01). Similarly, lower eGFR and lower BMI predicted higher CA3 concentrations (eGFR: standardized β = 0.24, *p* < 0.001; BMI: standardized β = 0.19, *p* < 0.0001). These findings support an association between lower estimated CI and higher skeletal muscle stress biomarkers, while also indicating that these concentrations are associated with renal function and BMI.

### 2.3. Venous Congestion Is Associated with Higher Skeletal Muscle Stress Biomarkers

Because venous congestion is a key pathophysiologic feature of AHF, we incorporated right atrial pressure (RAP) as a noninvasive surrogate of systemic venous loading. Using RAP ≥ 8 mmHg as the congestion threshold (Table 3), we classified patients into RAP < 8 and RAP ≥ 8 mmHg. The groups were comparable in anthropometric, hemodynamic, and routine laboratory parameters. However, patients with RAP ≥ 8 mmHg had higher CA3 (*p* = 0.040) and CK-MM (*p* = 0.028; Figure 1f,g), indicating greater skeletal-muscle stress in patients with higher estimated venous congestion. Myostatin, follistatin, and lactate did not differ by RAP category (Figure 1h–j).

### 2.4. Skeletal Muscle Stress Biomarkers Across Forrester-like Perfusion–Congestion Profiles

To relate hemodynamics to skeletal muscle biology more comprehensively, we applied a Forrester-style profile that combines perfusion (using CI) and congestion (using RAP). This framework captures the combined effects of low forward flow and venous loading, which may not be fully reflected by either parameter alone. Using CI < 2.2 L·min^−1^·m^−2^ to indicate low perfusion and RAP ≥ 8 mmHg to indicate congestion, we defined four hemodynamic subsets: ↓CI/↓RAP (*n* = 1; “cold–dry,” hypovolemic/underfilled), ↓CI/↑RAP (*n* = 14; “cold–wet,” hypoperfusion with congestion), ↑CI/↑RAP (*n* = 14; “warm–wet,” congestion with preserved output), and ↑CI/↓RAP (*n* = 6; “warm–dry,” high output with low venous pressure). Because the ↓CI/↓RAP group contained a single patient, it was excluded from between-group comparisons.

**Figure 2:**
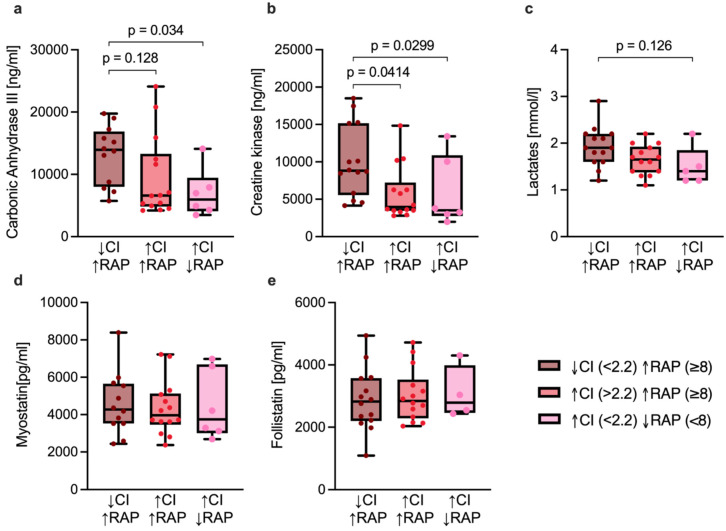
Skeletal muscle stress biomarkers and myokine signals across Forrester-style hemodynamic profiles (CI/RAP). (**a**–**e**) Plasma concentrations stratified by combined perfusion–congestion status: ↓CI/↑RAP (“cold–wet”), ↑CI/↑RAP (“warm–wet”), and ↑CI/↓RAP (“warm–dry–like”); the ↓CI/↓RAP profile was excluded (*n* = 1). Panels: (**a**) carbonic anhydrase III (CA3), (**b**) creatine kinase–MM (CK-MM), (**c**) lactate, (**d**) myostatin, and (**e**) follistatin. Boxes denote interquartile rang-es; central lines indicate medians; whiskers show minimum and maximum values; individual data points are displayed. Group comparisons used Kruskal–Wallis tests with Dunn’s post-hoc analysis. CI, cardiac index; RAP, right atrial pressure. ↑CI, CI > 2.2 L·min^−1^·m^−2^; ↓CI, CI <2.2 L·min^−1^·m^−2^; ↑RAP, RAP ≥ 8 mmHg, ↓RAP, RAP < 8 mmHg.

The skeletal muscle injury markers CA3 and CK-MM were highest in the cold–wet (↓CI/↑RAP) group (global *p* = 0.022 and *p* = 0.0094, respectively). Specifically, CA3 was higher in the cold–wet (↓CI/↑RAP) group than in warm–dry (↑CI/↓RAP; *p* = 0.034) and showed a trend toward being higher than warm–wet (↑CI/↑RAP; *p* = 0.128; Figure 2a). CK-MM was higher in cold–wet versus warm–wet (*p* = 0.0414) and higher versus warm–dry (*p* = 0.0299; Figure 2b). For both CA3 and CK-MM, the warm–wet and warm–dry profiles did not differ. Lactate showed a similar pattern, trending higher in the cold–wet group than in the other two profiles, with no difference between warm–wet and warm–dry (global *p* ≈ 0.06; Figure 2c). Myostatin and follistatin levels were similar across all profiles (Figure 2d,e). Together, these findings suggest that the combined presence of lower estimated perfusion and greater estimated venous congestion is associated with the highest circulating skeletal muscle stress biomarker concentrations in AHF.

### 2.5. High Serum Myostatin and Follistatin Levels During Episodes of AHF Normalize During Clinical Recovery

**Figure 3:**
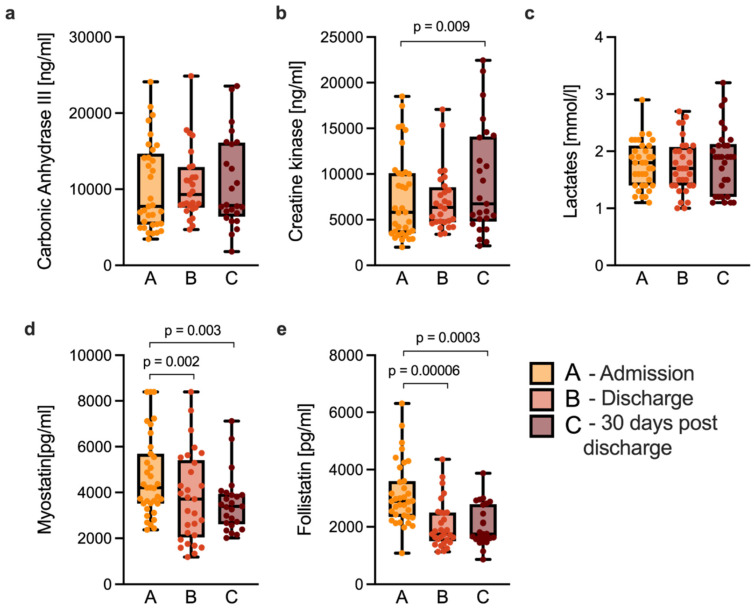
Skeletal muscle stress biomarkers and myokine signals across the clinical course of acute heart failure (admission, discharge, 30 days). Panels display serial plasma measurements at three time points, namely A (admission), B (discharge), and C (30-day follow-up): (**a**) carbonic anhydrase III (CA3), (**b**) creatine kinase–MM (CK-MM), (**c**) lactate, (**d**) myostatin, and (**e**) follistatin. Boxes show interquartile ranges, central lines indicate medians, whiskers represent minimum and maximum, and individual data points are plotted. Within-subject differences were assessed using repeated-measures ANOVA or the Friedman test.

To assess temporal changes during decompensation and early recovery, we measured circulating markers of skeletal muscle stress and regulation at admission (“A”), discharge (“B”), and 30-day follow-up (“C”). CA3 remained stable across admission, discharge, and 30-day follow-up (Figure 3a). CK-MM was similar between admission and discharge, then rose modestly by 30 days compared with admission (*p* = 0.009; Figure 3b). Lactate levels showed no clear change over time, with levels at discharge and follow-up comparable with those at admission (Figure 3c). In contrast, myostatin and its antagonist follistatin were highest at admission and were significantly lower at discharge and at 30 days (all *p* < 0.01; Figure 3d,e). These myokine trajectories are consistent with a transient response to acute decompensation that potentially improves with clinical stabilization.

## 3. Discussion

AHF is characterized by abrupt changes in forward flow and filling pressures that can affect organs beyond the heart. Peripheral hypoperfusion and venous congestion are recognized contributors to end-organ dysfunction [6], but the extent to which skeletal muscle is involved during acute decompensation remains incompletely understood. In this study, we evaluated whether circulating markers of skeletal muscle stress and catabolic regulation are associated with the acute hemodynamic profile in men hospitalized with acute heart failure.

We quantified CK-MM and CA3 as biomarkers of myofiber stress [10,11,12,13] and used noninvasive estimates of perfusion adequacy and systemic venous congestion, reflected by CI and RAP, to characterize the acute hemodynamic profile [19,20]. Both hemodynamic components were associated with skeletal muscle stress biomarkers. Lower CI was associated with higher CA3 and CK-MM concentrations, suggesting a relationship between reduced forward flow and greater skeletal muscle stress [6,10,11]. Similarly, patients with higher RAP had higher CA3 and CK-MM concentrations, consistent with the concept that venous hypertension may increase interstitial pressure and contribute to peripheral tissue stress during decompensation [21,22]. When CI and RAP were considered together, the ↓CI/↑RAP “cold–wet” profile showed the highest biomarker levels, suggesting that reduced estimated perfusion and elevated venous pressure may jointly contribute to a greater skeletal muscle stress signal [19,20]. Lactate showed a similar direction and is a recognized marker of impaired oxidative metabolism and adverse outcomes in AHF [10,11]. Although CK-MM and CA3 are commonly used as markers of skeletal muscle stress and remodeling, their circulating concentrations may also be shaped by renal function, body composition, and overall illness severity. In our cohort, patients with lower CI also had lower eGFR and lower BMI, indicating that higher CA3 and CK-MM should not be attributed exclusively to impaired muscle perfusion. Reduced eGFR may also reflect acute cardiorenal dysfunction during decompensation and may occur alongside skeletal muscle stress as part of the broader systemic response to AHF. Thus, rather than representing isolated variables, hemodynamic impairment, renal dysfunction, body composition, and skeletal muscle stress likely reflect interconnected features of acute decompensation.

In normal conditions, muscle-secreted myokines coordinate skeletal muscle adaptation to physiological stimuli, including physical activity [23]. During AHF, however, altered myokine signaling may reflect a shift toward muscle stress, catabolic remodeling and impaired recovery. Myostatin is a potent inhibitor of skeletal muscle anabolism that promotes proteolysis and catabolic signaling [17]. In our cohort, lower perfusion was associated with higher circulating myostatin, consistent with reports linking elevated myostatin to HF severity [16,17]. Myostatin was lower 30 days after clinical stabilization than during acute decompensation, suggesting that this catabolic muscle signaling may be more pronounced during AHF and attenuate with recovery. Follistatin showed a similar trajectory, potentially reflecting a compensatory or counter-regulatory response to myostatin activation. Interestingly, elevated follistatin has also been associated with adverse outcomes in HF [14,15]. CA3 and lactate did not significantly decline during follow-up, and CK-MM was modestly higher at 30 days, which could also relate to delayed muscle recovery, remobilization after discharge, altered clearance, or persistent systemic stress. Because this study did not include a non-AHF control group, these longitudinal changes should be interpreted as relative within-patient patterns rather than evidence of absolute biomarker elevation during AHF.

From a clinical perspective, these findings suggest that skeletal muscle-related biomarkers may help characterize peripheral involvement during AHF and identify patients in whom acute decompensation is accompanied by greater skeletal muscle stress. Although our results do not establish causality or define a therapeutic target, they support future studies testing whether muscle-focused assessments, early mobilization, rehabilitation, nutritional support, or interventions targeting muscle metabolism and catabolic signaling may improve recovery after AHF hospitalization. Future mechanistic and translational studies should integrate hemodynamic assessment with direct measures of skeletal muscle structure and function to determine whether skeletal muscle stress is a modifiable component of AHF recovery.

This study comes with a few important limitations. First, it was a single-center pilot study with a relatively small sample size, which limits its statistical power, particularly for subgroup analyses and multivariable modeling. Therefore, the findings should be interpreted as exploratory and hypothesis-generating. Second, only male patients were included to reduce biological variability in this small cohort. However, this limits the generalizability of the results, and future studies should include both women and men. Third, CI and RAP were estimated by using noninvasive methods rather than invasive hemodynamic measurements, which remain the gold standard. Although these bedside approaches are clinically practical and allow hemodynamic assessment in a broader group of patients without the risks of catheterization [24], they provide estimates rather than direct measurements and may be less precise. Finally, elevated skeletal muscle-related biomarkers in AHF may reflect not only hemodynamic impairment, but also renal function, body composition, and systemic illness severity. Larger studies using invasive hemodynamic assessment and adequately powered multivariable analyses are needed to confirm our findings.

## 4. Material and Methods

### 4.1. Patients

**Figure 4:**
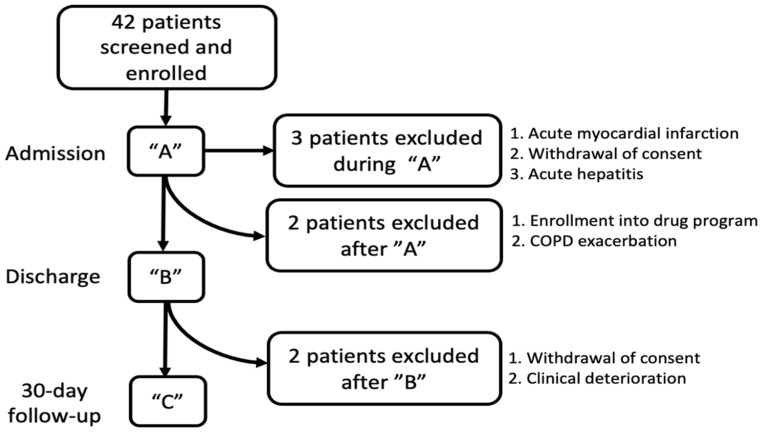
Study flow and visit schedule. Forty-two patients with acute heart failure were enrolled and evaluated at three timepoints: A, admission; B, discharge; and C, 30-day follow-up. During Timepoint A, 3 patients were excluded (acute myocardial infarction, withdrawal of consent, acute hepatitis). Between Timepoints A and B, 2 patients were excluded (enrollment in another drug program, COPD exacerbation). Between Timepoints B and C, 2 patients were excluded (withdrawal of consent, clinical deterioration).

The study included 42 selected patients hospitalized at the Centre for Heart Diseases, 4th Military Hospital, Wroclaw, Poland, due to AHF. The study protocol was approved by the local Ethics Committee, and the study was conducted in compliance with the Helsinki Declaration. This study has been registered with Clinicaltrials.gov (NCT03102164). The entry criteria included (a) age ≥ 18 years old; (b) written informed consent to participate in the study; (c) hospitalization due to acute heart failure; (d) clinical signs of congestion, including persistent dyspnea at rest or with minimal exertion at screening and at the time of enrolment, pulmonary rales, peripheral oedema and pulmonary congestion on chest radiograph (all needed to be present); and (e) elevated N-terminal pro-B-type natriuretic peptide (NTproBNP) ≥ 2000 pg/mL. AHF was defined according to the current 2021 ESC Guidelines [3] as a rapid or gradual onset of symptoms and/or signs of heart failure requiring unplanned hospitalization of the patient. Exclusion criteria included (a) acute coronary syndrome according to the ESC’s definition; (b) bacterial infection; (c) pre-existing chronic respiratory failure; (d) requirement for mechanical invasive ventilation; (e) significant arrhythmia (arrhythmia that was hemodynamically unstable and/or needed urgent targeted treatment); (f) anemia (hemoglobin < 9 g/dL); (g) active neoplastic process; (h) chronic kidney failure with creatinine clearance < 30 mL/min; (i) acute deep vein thrombosis and significant orthopedic diseases of the lower limbs; and/or (j) mental or cognitive conditions that precluded participation in the study. Study assessments were conducted at three timepoints (Figure 4): (A) admission (Days 1 and 2 of hospitalization for acute heart failure), (B) hospital discharge, and (C) 30 days post-discharge after confirmation of clinical stability [3].

### 4.2. Standard Laboratory Parameters

The following laboratory parameters were assessed using standard methods in the hospital laboratory: hemoglobin as a clinical marker of anemia; capillary blood gas and acid–base parameters, including oxygen saturation (%); oxygen partial pressure (mmHg), and lactate (mmol/L), measured using the ABL 800 Flex analyzer (Radiometer, Copenhagen, Denmark); and renal function indices, including serum creatinine (mg/dL) and the estimated glomerular filtration rate (eGFR). eGFR was calculated using the Modification of Diet in Renal Disease (MDRD) formula: eGFR = 175 × serum creatinine^−1.154^ × age^−0.203^. Markers of cardiac stress and injury included plasma NTproBNP, measured by immunoenzymatic assay (Siemens, Marburg, Germany), and troponin I, measured by immunoenzymatic assay using the Dimension RxL Max system (Siemens).

### 4.3. Immunoassays for Markers of Skeletal Muscle Homeostasis and Muscle Stress

Biomarkers of skeletal muscle stress were assessed from peripheral blood. Serum follistatin (pg/mL) and myostatin (GDF-8, pg/mL) were measured using a commercially available enzyme-linked immunosorbent assay (R&D Systems, Minneapolis, MN, USA). Serum Carbonic Anhydrase III (muscle-specific; CA3, ng/mL) and creatin kinase (muscle-specific; CK-MM, ng/mL) assessed by enzyme-linked immunosorbent assay (Novus Biologicals, Centennial, CO, USA). For all ELISA tests, we performed a dilution series of serum samples in order to obtain absorption values at the middle of the standard curve. Finally, the following dilutions were chosen as the most appropriate: 2-fold for follistatin, 4-fold for myostatin, 800-fold for CA3, and 850-fold for CK-MM. All measurements were performed in plasma collected at (A) Days 1 and 2 of hospitalization, (B) the last day of hospitalization or discharge from hospital, and (C) at 30 days of clinical recovery (after discharge). ELISA outliers were excluded using the ROUT method (Q = 1%) [25].

### 4.4. Transthoracic Echocardiography

Transthoracic echocardiography (GE Vivid) was performed in all participants following EACVI recommendations. Left ventricular ejection fraction (LVEF) was measured by the biplane Simpson method. Right atrial pressure (RAP) was estimated using inferior vena cava diameter and inspiratory collapsibility [26]. RAP was assigned according to standard categories as 3, 8, or 15 mmHg, reflecting low, intermediate, or elevated estimated RAP, respectively. Because echocardiographic RAP estimation provides a stepwise rather than truly continuous measure, RAP was analyzed categorically for subgroup analyses using a prespecified threshold of RAP ≥ 8 mmHg to define elevated RAP.

### 4.5. Transthoracic Impedance Cardiography

Transthoracic impedance cardiography (ICG) was used to assess central hemodynamics within the first 48 h of hospitalization (PhysioFlow Enduro, Manatec Biomedical, Paris, France). Recordings were obtained during a 15-min, supine, resting acquisition with six pre-gelled electrodes placed per manufacturer guidance: two on the left lateral neck, two precordial for ECG, and two near the xiphisternum. The system measures baseline thoracic impedance (Z_0_), the first derivative of impedance (dZ/dt)-derived velocity and acceleration indices, ventricular ejection time, heart rate, and an early diastolic filling ratio, from which stroke volume (SV), cardiac output (CO), and cardiac index (CI = CO/body surface area) were computed. For analysis, CI values were averaged over the recording; a CI threshold of 2.2 L·min^−1^·m^−2^ was prespecified to indicate hypoperfusion (low output) for subgrouping in accordance with Forrester-style profiling.

### 4.6. Statistical Analysis

Continuous variables with a normal distribution (following confirmation with a Levene test and Shapiro–Wilk test for the distribution and homogeneity of the variance) were described using means  ±  standard deviation, whereas variables with skewed distribution were described by medians with upper and lower quartiles, and categorized variables were given as numbers and percentages. The relations between myokines and clinical parameters were analyzed using Spearman’s rank correlation. The non-parametric Mann–Whitney U test was used to compare differences between two independent groups of patients with CI < 2.2 and CI > 2.2 L·min^−1^·m^−2^ or RAP < 8 and RAP ≥ 8 mmHg. Exploratory multiple linear regression analyses were performed to identify the predictors of circulating CK-MM and CA3 concentrations, and candidate variables were selected on the basis of clinical relevance and univariate findings. Forrester-style hemodynamic profile (CI/RAP) comparisons used Kruskal–Wallis tests with Dunn’s post hoc analysis with adjustment for multiple testing. The repeated measurements analysis of variance (ANOVA) and Friedman’s test were used to calculate the difference between myokine concentrations at 3 timepoints (“A”, “B”, and “C”. A *p*-value of < 0.05 was considered statistically significant. Statistical analyses were performed using the STATISTICA 13 (StatSoft, StatSoft Polska Sp. z o.o., Kraków, Poland). Graphs were generated in GraphPad Prism 10 (GraphPad Software, Boston, MA, USA).

## 5. Conclusions

This study suggests that skeletal muscle may be a vulnerable peripheral tissue during acute heart failure. Lower estimated perfusion and greater venous congestion were associated with higher circulating markers of skeletal muscle stress, suggesting that these biomarkers may reflect skeletal muscle involvement during acute decompensation. These exploratory findings support further investigation of skeletal muscle stress biomarkers in AHF and highlight the need for larger studies integrating hemodynamic assessment with direct measures of skeletal muscle structure and function.

## Figures and Tables

**Table 1:** Baseline characteristics of AHF patients.

Clinical Variable	Men with Acute Heart Failure AHF, *n* = 35
Age (years)	66 ± 11
BMI (kg/m^2^)	29.7± 6.4
AHF etiology, ischemic (*n*, %)	19 (54)
HFrEF (*n*, %)	24 (69)
HFmEF (*n*, %)	6 (18)
HFpEF (*n*, %)	5 (14)
LVEF (%)	33.8 ± 11
LVEDD (%)	64 ± 11.4
sBP (mmHg)	121 ± 21
SpO_2_ (%)	95%
PaO_2_ (mmHg)	67 ± 11
Lac (mmol/L)	1.8 (1.4–2.1)
NTproBNP (pg/mL)	6522 ± 4750
Troponin (μg/L)	0.038 (0.023–0.05)
Hemoglobin (g/dL)	13.2 ± 2
Creatinine (mg/dL)	1.5 ± 0.8
Uric Acid (mg/dL)	8.9 ± 2.7
eGFR (mL/min/1.73 m^2^)	69 (47–97)
Comorbidities:	
Hypertension (*n*, %)	21 (60%)
Diabetes mellitus (*n*, %)	18 (51%)
COPD (*n*, %)	6 (18%)
ICD (*n*, %)	9 (27%)
CRT-D (*n*, %)	5 (15%)
CABG (*n*, %)	4 (12%)
AF (*n*, %)	22 (63%)
QRS > 120 (*n*, %)	13 (38%)
LBBB (*n*, %)	5 (15%)
Pharmacological treatment:	
ACE-I and/or ARB (*n*, %)	30 (86%)
Beta-blocker (*n*, %)	35 (100%)
MRAs (*n*, %)	15 (43%)
Digoxin (*n*, %)	3 (9%)
Loop diuretics (*n*, %)	35 (100%)
Vasodilator (*n*, %)	24 (69%)
Dobutamine (*n*, %)	1 (3%)
Noradrenaline (*n*, %)	0 (0%)

BMI, body mass index; HFrEF, heart failure with reduced left, HFmEF, heart failure with mildly reduced left ventricular ejection fraction; HFpEF, heart failure with preserved left ventricular ejection fraction; LVEF, left ventricular ejection fraction; LVEDD, left ventricular end-diastolic diameter; sBP, systolic blood pressure; SpO_2_, blood oxygen saturation; PaO_2_, partial pressure of oxygen; Lac, lactate; NTproBNP, N-terminal pro-B-type natriuretic peptide; eGFR, estimated glomerular; ICD, implantable cardioverter-defibrillator; CRT, cardiac resynchronization therapy; CABG, coronary artery bypass graft; AF, atrial fibrillation; LBBB, left bundle branch block; ACE-I, angiotensin-converting enzyme inhibitor; ARB, angiotensin receptor blocker; MRAs, mineralocorticoid receptor antagonists.

**Table 2:** Clinical characteristics by cardiac index category.

Clinical Variable	Cardiac Index CI ≤ 2.2 (*n* = 15)	Cardiac Index CI > 2.2 (*n* = 20)	*p*-Value
	Median (Q1–Q3)	Median (Q1–Q3)	
Age (years)	69 (57–76)	65 (55–69)	0.29
BMI (kg/m^2^)	26.6 (23–29)	31.6 (26–38)	**0.03**
HR (bpm)	85 (60–114)	79 (56–124)	0.89
SBP (mmHg)	108 (100–110)	125 (115–140)	**0.03**
DBP (mmHg)	65 (60–71)	78 (70–90)	**0.006**
Troponin (ng/mL)	0.039 (0.023–0.0014)	0.038 (0.024–0.046)	0.47
NTproBNP (pg/mL)	5525 (3099–9200)	3879 (3217–7189)	0.78
LVEF (%)	27 (25–45)	27	0.85
RAP (mmHg)	15 (15–15)	15 (3–15)	0.15
Hemoglobin (g/dL)	13.2 (12–14)	14.9 (12–15)	**0.04**
Creatinine (mg/dL)	1.5 (1.3–1.8)	1.1 (1.0–1.2)	**0.001**
eGFR (mL/min/1.73 m^2^)	48 (37–60)	94 (68–108)	**0.0001**

BMI, body mass index; HR, heart rate; SBP/DBP, systolic/diastolic blood pressure; Troponin, cardiac troponin I; NTproBNP, N-terminal pro-B-type natriuretic peptide; LVEF, left ventricular ejection fraction; RAP, right atrial pressure; Creatinine, serum creatinine; eGFR, estimated glomerular filtration rate (mL·min^−1^·1.73 m^−2^). Continuous variables are presented as medians (interquartile range). *p*-values reflect between-group comparisons (Mann–Whitney U test). Bolded *p*-values are statisticaly significant.

**Table 3:** Clinical characteristics by right atrial pressure (RAP) category.

Clinical Variable	Right Atrial Pressure RAP < 8 (*n* = 6)	Right Atrial Pressure RAP ≥ 8 (*n* = 29)	*p*-Value
	Median (Q1–Q3)	Median (Q1–Q3)	
Age (years)	58 (52–68)	67 (58–72)	0.29
BMI (kg/m^2^)	26.4 (23–36)	29.2 (25–33)	0.48
HR (bpm)	87 (80–91)	85 (64–95)	0.70
SBP (mmHg)	124 (101–140)	110 (102–135)	0.59
DBP (mmHg)	82 (66–90)	70 (62–79)	0.24
Troponin (ng/mL)	0.04 (0.03–0.05)	0.04 (0.02–0.05)	0.64
NTproBNP (pg/mL)	5644 (3829–10346)	4082 (3081–7658)	0.36
LVEF (%)	30 (25–30)	29 (25–45)	0.79
CI (L/min/m^2^)	2.8 (2.5–3.0)	2.3 (2–2.9)	0.08
Hemoglobin (g/dL)	14.9 (14–15)	13.4 (12–14)	0.15
Creatinine (mg/dL)	1.1 (0.92–1.1)	1.3 (1.1–1.8)	0.07
eGFR (ml/min/1.73 m^2^)	102 (69–122)	62 (39–90)	0.09

BMI, body mass index; HR, heart rate; SBP/DBP, systolic/diastolic blood pressure; Troponin, cardiac troponin I; NTproBNP, N-terminal pro-B-type natriuretic peptide; LVEF, left ventricular ejection fraction; CI, cardiac index; Creatinine, serum creatinine; eGFR, estimated glomerular filtration rate (mL·min^−1^·1.73 m^−2^). Patients were stratified by echocardiographic RAP into RAP < 8 mmHg (*n* = 6) and RAP ≥ 8 mmHg (*n* = 29). Continuous variables are presented as median (interquartile range). *p*-values reflect between-group comparisons (Mann–Whitney U test).

## Data Availability

The data supporting the findings of this study are available from the corresponding author upon reasonable request.

## References

[1] Rossello X., Miró Ò., Llorens P., Jacob J., Herrero-Puente P., Gil V., Espiga F.R., Romero R., Vidán M.T., Bueno H. (2019). Effect of Barthel Index on the Risk of Thirty-Day Mortality in Patients with Acute Heart Failure Attending the Emergency Department: A Cohort Study of Nine Thousand Ninety-Eight Patients From the Epidemiology of Acute Heart Failure in Emergency Departments Registry. Ann. Emerg. Med..

[2] Takara Y., Saitoh M., Morisawa T., Takahashi T., Yoshida N., Sakiyama M., Nakamura R., Tei I., Fujiwara T. (2021). Clinical Characteristics of Older Heart Failure Patients with Hospital-Acquired Disability: A Preliminary, Single-Center, Observational Study. Cardiol. Res..

[3] McDonagh T.A., Metra M., Adamo M., Gardner R.S., Baumbach A., Böhm M., Burri H., Butler J., Čelutkienė J., Chioncel O. (2022). 2021 ESC Guidelines for the diagnosis and treatment of acute and chronic heart failure: Developed by the Task Force for the diagnosis and treatment of acute and chronic heart failure of the European Society of Cardiology (ESC) with the special contribution of the Heart Failure Association (HFA) of the ESC. Eur. J. Heart Fail..

[4] Savarese G., Becher P.M., Lund L.H., Seferovic P., Rosano G.M.C., Coats A.J.S. (2023). Global burden of heart failure: A comprehensive and updated review of epidemiology. Cardiovasc. Res..

[5] Njoroge J.N., Teerlink J.R. (2021). Pathophysiology and Therapeutic Approaches to Acute Decompensated Heart Failure. Circ. Res..

[6] Harjola V.-P., Mullens W., Banaszewski M., Bauersachs J., Brunner-La Rocca H.P., Chioncel O., Collins S.P., Doehner W., Filippatos G.S., Flammer A.J. (2017). Organ dysfunction, injury and failure in acute heart failure: From pathophysiology to diagnosis and management. A review on behalf of the Acute Heart Failure Committee of the Heart Failure Association (HFA) of the European Society of Cardiology (ESC). Eur. J. Heart Fail..

[7] Zymliński R., Sokolski M., Biegus J., Siwołowski P., Nawrocka-Millward S., Sokolska J.M., Dudkowiak M., Marciniak D., Todd J., Jankowska E.A. (2019). Multi-organ dysfunction/injury on admission identifies acute heart failure patients at high risk of poor outcome. Eur. J. Heart Fail..

[8] Hamatani Y., Iguchi M., Ikeyama Y., Kunugida A., Ogawa M., Yasuda N., Fujimoto K., Ichihara H., Sakai M., Kinoshita T. (2022). Prevalence, Temporal Change, and Determinants of Anxiety and Depression in Hospitalized Patients with Heart Failure. J. Card. Fail..

[9] Fonarow G.C., Stough W.G., Abraham W.T., Albert N.M., Gheorghiade M., Greenberg B.H., M O’Connor C., Sun J.L., Yancy C.W., Young J.B. (2007). Characteristics, treatments, and outcomes of patients with preserved systolic function hospitalized for heart failure: A report from the OPTIMIZE-HF Registry. J. Am. Coll. Cardiol..

[10] Bosso G., Mercurio V., Diab N., Pagano A., Porta G., Allegorico E., Serra C., Guiotto G., Numis F.G., Tocchetti C.G. (2021). Time-weighted lactate as a predictor of adverse outcome in acute heart failure. ESC Heart Fail..

[11] Zymliński R., Biegus J., Sokolski M., Siwołowski P., Nawrocka-Millward S., Todd J., Jankowska E.A., Banasiak W., Cotter G., Cleland J.G. (2018). Increased blood lactate is prevalent and identifies poor prognosis in patients with acute heart failure without overt peripheral hypoperfusion. Eur. J. Heart Fail..

[12] Brancaccio P., Lippi G., Maffulli N. (2010). Biochemical markers of muscular damage. Clin. Chem. Lab. Med..

[13] Väänänen H.K., Takala T.E., Tolonen U., Vuori J., Myllyla V.V. (1988). Muscle-specific carbonic anhydrase III is a more sensitive marker of muscle damage than creatine kinase in neuromuscular disorders. Arch. Neurol..

[14] Tanaka K., Valero-Muñoz M., Wilson R.M., Essick E.E., Fowler C.T., Nakamura K., Hoff M.v.D., Ouchi N., Sam F. (2016). Follistatin like 1 Regulates Hypertrophy in Heart Failure with Preserved Ejection Fraction. JACC Basic Transl. Sci..

[15] Pan J., Nilsson J., Engström G., De Marinis Y. (2024). Elevated circulating follistatin associates with increased risk of mortality and cardiometabolic disorders. Nutr. Metab. Cardiovasc. Dis..

[16] Lenk K., Erbs S., Höllriegel R., Beck E., Linke A., Gielen S., Winkler S.M., Sandri M., Hambrecht R., Schuler G. (2012). Exercise training leads to a reduction of elevated myostatin levels in patients with chronic heart failure. Eur. J. Prev. Cardiol..

[17] Gruson D., Ahn S.A., Ketelslegers J.-M., Rousseau M.F. (2011). Increased plasma myostatin in heart failure. Eur. J. Heart Fail..

[18] Forrester J.S., Diamond G.A., Swan H.J. (1977). Correlative classification of clinical and hemodynamic function after acute myocardial infarction. Am. J. Cardiol..

[19] Chioncel O., Mebazaa A., Maggioni A.P., Harjola V., Rosano G., Laroche C., Piepoli M.F., Crespo-Leiro M.G., Lainscak M., Ponikowski P. (2019). Acute heart failure congestion and perfusion status—Impact of the clinical classification on in-hospital and long-term outcomes; insights from the ESC-EORP-HFA Heart Failure Long-Term Registry. Eur. J. Heart Fail..

[20] Takahashi T., Iwano H., Shibayama K., Kitai T., Tanaka H., Yamada H., Sata M., Kusunose K. (2023). The Clinical Utility of Noninvasive Forrester Classification in Acute Heart Failure from PREDICT Study. Am. J. Cardiol..

[21] Dupont M., Mullens W., Tang W.H.W. (2011). Impact of systemic venous congestion in heart failure. Curr. Heart Fail. Rep..

[22] Ganda A., Onat D., Demmer R.T., Wan E., Vittorio T.J., Sabbah H.N., Colombo P.C. (2010). Venous Congestion and Endothelial Cell Activation in Acute Decompensated Heart Failure. Curr. Heart Fail. Rep..

[23] Hoffmann C., Weigert C. (2017). Skeletal Muscle as an Endocrine Organ: The Role of Myokines in Exercise Adaptations. Cold Spring Harb. Perspect. Med..

[24] Kim K.-H., Jentzer J.C., Wiley B.M., Miranda W.R., Bennett C., Barsness G.W., Oh J.K. (2021). Diamond-Forrester classification using echocardiography haemodynamic assessment in cardiac intensive care unit patients. ESC Heart Fail..

[25] Motulsky H.J., Brown R.E. (2006). Detecting outliers when fitting data with nonlinear regression—A new method based on robust nonlinear regression and the false discovery rate. BMC Bioinform..

[26] Mukherjee M., Rudski L.G., Addetia K., Afilalo J., D’aLto M., Freed B.H., Friend L.B., Gargani L., Grapsa J., Hassoun P.M. (2025). Guidelines for the Echocardiographic Assessment of the Right Heart in Adults and Special Considerations in Pulmonary Hypertension: Recommendations from the American Society of Echocardiography. J. Am. Soc. Echocardiogr..

